# Facultative mutualism between *Paramecium* and the intracellular *Rickettsiales* bacterium *Megaera* mediated by a horizontally acquired biotin operon

**DOI:** 10.1093/ismeco/ycag079

**Published:** 2026-03-27

**Authors:** Michele Giovannini, Leandro Gammuto, Tania Alonso-Vásquez, Natalia Gogoleva, Greta Bellinzona, Alexey Potekhin, Giulio Petroni, Michele Castelli

**Affiliations:** Department of Biology, University of Pisa, Via Volta 4/6, 56100, Pisa, PI, Italy; Department of Biology, University of Florence, Via Madonna del Piano, 6, 50019 Sesto Fiorentino, Florence, Italy; Department of Biology and Biotechnology, University of Pavia, Via Ferrata 9, 27100 Pavia, PV, Italy; Department of Biology, University of Florence, Via Madonna del Piano, 6, 50019 Sesto Fiorentino, Florence, Italy; Research Department for Limnology, University of Innsbruck, Mondseestrasse 9A-5310 Mondsee, Austria; Department of Biology and Biotechnology, University of Pavia, Via Ferrata 9, 27100 Pavia, PV, Italy; Research Department for Limnology, University of Innsbruck, Mondseestrasse 9A-5310 Mondsee, Austria; Department of Biology, University of Pisa, Via Volta 4/6, 56100, Pisa, PI, Italy; Department of Biology and Biotechnology, University of Pavia, Via Ferrata 9, 27100 Pavia, PV, Italy; Department of Biology, University of Pisa, Via Volta 4/6, 56100, Pisa, PI, Italy

**Keywords:** dual RNA-Seq, *Rickettsiales*, ciliate protists, biotin, nutritional mutualism, bacterial endosymbiont, phylogeny, comparative genomics transcriptomics, bacteria–host interactions, *Wolbachia*

## Abstract

The *Rickettsiales* are an alphaproteobacterial lineage engaging in ancient associations with a variety of eukaryotic hosts and with a wide spectrum of effects. They include vector-borne pathogens, as well as *Wolbachia*, which ranges from a reproductive manipulator to a mutualist in arthropods and nematodes. The majority of *Rickettsiales* are associated with aquatic protists, but these interactions are poorly understood. Here, we explored by dual RNA-Seq the effect of the host-generalist *Rickettsiales* bacterium *Megaera polyxenophila* on the protist *Paramecium primaurelia*. *Megaera* induces substantial changes in host gene expression, in particular, increased expression levels of certain cell replication–related functions, consistent with the higher growth observed in previous experiments. Conversely, the co-occurring reduction of catabolism and energy metabolism can be explained by the capability of the bacterium to efficiently exert the same pathways also for the host’s benefit. Therefore, *Megaera* likely behaves as a facultative mutualist, consistent with its predicted ability to provide the host with Adenosine triphosphate (ATP) and biotin, the latter synthesized, thanks to a recently horizontally acquired operon. At the same time, this bacterium expresses several genes involved in host cell invasion and possibly toxicity. Accordingly, it is envisioned that the overall effect of *Megaera* on its host is rather plastic, being the fine-tuned sum of supportive and parasitic actions, likely resulting in flexibility according to host and symbiont genotypes and environmental conditions, and subjected to evolutionary changes. Such flexibility may also explain the broad host range of *Megaera* and, from a more general perspective, hints for shared traits and analogies among other protist-associated *Rickettsiales*.

## Introduction

The *Rickettsiales* are a deep alphaproteobacterial lineage with an ancient association with eukaryotes [1, 2]. Their best studied representatives are arthropod-borne pathogens [3–6] and *Wolbachia*, hosted by arthropods and nematodes [7–10]. On the other hand, the still under-explored majority of known *Rickettsiales* are associated with protists, including amoebas, ciliates, algae, and other aquatic organisms, such as cnidarians [11–20]. Investigations on those aquatic members have been extremely useful for enlightening evolutionary trends among the *Rickettsiales*, including the emergence of traits typical of the pathogenic representatives [1, 2, 6, 21–23]. However, with only a few partial exceptions [13, 24, 25], these investigations typically consisted of a combination of microscopy observations and comparative genomics, without direct experimental data on how these bacteria interact with their hosts and on the reciprocal effects.

Here, we focused, as a model organism, on the *Rickettsiales* bacterium ‘*Candidatus* Megaera polyxenophila’ (formerly ‘*Candidatus* Megaira polyxenophila’ [26], from here on, *Megaera polyxenophila*), a widespread *Rickettsiaceae* bacterium, particularly in freshwater [27]. This species is closely related to the well-studied pathogen *Rickettsia*, and is found in diverse hosts such as ciliates, streptophytes, chlorophytes, ochrophytes, and cercozoans [15, 20, 27–32]. The host range of the *Megaera* genus is even broader, encompassing also hydras, corals, ascidians, and haplosporidians [33–36].

While the interaction modes and mechanisms between *Megaera* and its hosts are poorly understood, the broad host range and the seemingly harmless long-term laboratory propagation of colonized hosts are suggestive of a mutualism/commensalism or a very specialized parasitism [37]. Exploratory experiments showed that, in laboratory conditions, symbiotic *Paramecium* ciliates reach higher cell densities than aposymbiotic lines, which can be indicative of some mutualistic action [25]. Genome data overall indicate a possible parasitic role, due to the massive scavenging of metabolic precursors, including intermediates of Krebs cycle [2], similarly to many other *Rickettsiales* [38]. On the other hand, some genome-encoded traits, particularly polyketide biosynthesis, could indicate a defensive symbiotic contribution [30].

Here, we performed a dual RNA-Seq (i.e. simultaneous bacterial and host transcriptome sequencing [39]) investigation on *Megaera* hosted in the cytoplasm of the freshwater ciliate *Paramecium primaurelia*, comparing symbiotic and aposymbiotic host lines.

Transcriptomics has been valuable in investigating bacterial–host interactions involving human pathogenic *Rickettsiales* [40, 41], as well as a few non-pathogenic others, including aquatic ones [13, 29, 42], and has also been employed on some occasions on symbionts of protists [43–47], including ciliates [48–50]. However, to our best knowledge, this is the first study to apply dual RNA-Seq to assess the impact of a *Rickettsiales* bacterium on a protist host.

## Materials and methods

### Strains employed and laboratory maintenance

In this work, we used previously established laboratory cultures of *P. primaurelia* Rio Lg_Jac 2III naturally harbouring *M. polyxenophila* (from here on, LgJac_Meg+), and a subclone of the same strain that had been treated with antibiotics to remove the bacterium (from here on, LgJac_Meg−) [25]. Strains were propagated at 19°C in the laboratory by regular feeding with Cerophyll medium inoculated with *Raoultella planticola* as previously described [25]. The presence/absence status of the symbiont was preliminarily confirmed by fluorescence *in situ* hybridization using the protocol by [51], as described previously [27].

### Sequencing and assembly of the *Megaera* genome

A whole-genome amplification (WGA) was performed on ~10 cells of *P. primaurelia* LgJac_Meg + as previously described [21]. The WGA product was processed by a TruSeq PCR-free library and sequenced with Illumina NovaSeq X by Macrogen Europe (Amsterdam, the Netherlands), generating 244 840 594 2 × 150 bp reads, as well as with Oxford Nanopore Technology (ONT) by Eurofins Genomics (Ebersberg, Germany) on a GridION machine, generating 755 646 921 bp output (219 911 reads, N50 = 4606 bp). Raw Illumina reads were then trimmed with Trimmomatic V0.39 [52], removing adapters and low-quality bases at both ends. The complete set of trimmed Illumina reads was assembled using SPAdes v3.6 [53]. The blobology pipeline [54] was employed to classify contigs based on GC content, sequencing coverage, and homology (Fig. S1), in order to help distinguish those belonging to *Megaera* from those of *Paramecium* and other bacterial species. Specifically, we selected the reads mapped with bowtie2 [55] on the contigs with coverage higher than 800, apart from those with the best megablast hit on *Paramecium* spp. These were separately reassembled with SPAdes, and the output was carefully manually curated to ensure an accurate assembly, as previously described (e.g. [21, 56]). Then, ONT reads were filtered with Filtlong v0.3.1 (https://github.com/rrwick/Filtlong) in two sequential steps, the first one without external reference and with --min_length 1000 –keep_percent 50 options, the second one using the Illumina assembly as reference, and the option –keep_percent 20. Then, a hybrid assembly was obtained from selected Illumina and ONT reads with Unicycler v0.5.0 [57] with the bold mode, followed by further manual refinement [2]. The resulting genome was annotated with Prokka v1.13.7 [58] with the --rfam option. The assembly quality and completeness were verified using BUSCO v6.0.0 [59] and the rickettsiaceae_odb12 reference dataset.

### Pangenome analysis of *M. polyxenophila*

Twenty-one genome assemblies assigned to *M. polyxenophila* were selected for the analysis, including LgJac and others downloaded from NCBI. All genomes were annotated using Prokka, and their assembly quality was assessed using BUSCO as described above, applying cutoffs (>80% complete and single-copy and <2% duplicated reference orthologs) to obtain a final selection of 13 genomes (Table S1). Those were used as input for a pangenomic analysis with PpanGGOLiN [60], utilizing the ‘all’ subcommand. PPanGGOLiN identifies gene families and classifies them into three partitions according to their presence/absence in the dataset, namely, from the most to the least common, persistent, shell, and cloud. To refine this classification, the persistent partition was further subdivided into core (present in all the genomes), and soft core (not present in all the genomes) subpartitions. The resulting four classes (core, soft core, shell, cloud) were employed for the transcriptome enrichment analysis (see below).

### Sample processing and dual transcriptome sequencing

The LgJac_Meg + and LgJac_Meg− *Paramecium* cultures were regularly fed (every second day) for three weeks with a proportional volume (0.25) of bacterized Cerophyll medium, then left unfed for 1 week. Then, they were cleaned and concentrated, adapting a previously established protocol [61]. For each culture, ~300 ml were sieved through a nylon filter (pore size 100 μm), to remove the bigger clumps of debris and food bacteria (Fig. S2). Subsequently, to obtain highly concentrated and clean cultures, three centrifugation steps (at 120 g for 10′) were performed with a Rotofix 46 (Hettich) centrifuge. After each step, the supernatant, containing small debris, was discarded, and the pellet, containing the paramecia, was re-suspended in sterile medium. At the end of the last step, each pellet was resuspended in a small residual amount of medium (~300 μl) and equally split into three parts, which were further processed and analysed separately, as replicates.

RNA extraction was performed using the RNeasy Mini kit (QIAGEN). Briefly, RTL buffer (600 μl) was added to each replicate, which was then homogenized using an RNAse-free syringe, and processed according to the manufacturer’s protocol, including DNAse I treatment. Extracted RNA was quantified with the Qubit RNA High Sensitivity assay (Thermo Fisher Scientific). Subsequently, each sample was equally split into two parts. The first part was processed for the host RNA-seq, namely through a TruSeq stranded mRNA library including the Ribo-Zero Plus treatment for rRNA removal, and sequenced on an Illumina NovaSeq 6000 Machine, with 2x100 bp reads (‘poly(A)-enriched reads’). The second part of each sample was processed for the symbiont RNA-seq, using the NEBNext Magnetic Oligo d(T)25 beads (New England Biolabs) to deplete from eukaryotic mRNA. The manufacturer’s instructions were followed up to the magnetic separation of the eukaryotic mRNA-bound beads from the supernatant, which contained other RNA species (including the targeted bacterial mRNA). Thus, RNA was recovered from the supernatant employing RNAClean XP beads (Beckman Coulter) following the manufacturer’s instructions. Then, the samples were subjected to TruSeq stranded total RNA libraries with Ribo-Zero treatment and sequenced as above (‘poly(A)-depleted reads’).

All library preparation (including Ribo-Zero treatments) and sequencing were carried out at Macrogen Europe.

### RNA-seq data analysis

The trimming procedure to eliminate adapter sequences present in our data was carried out by the sequencing company. The quality of the resulting reads was assessed using FastQC 0.11.7 [61]. For *P. primaurelia*, we downloaded from ParameciumDB (https://paramecium.i2bc.paris-saclay.fr/; accessed on 24 July 2024) the macronuclear genome, together with the respective annotation, to be used as reference. For *Megaera*, the *de novo* LgJac-assembled genome sequence annotated with Prokka was used as a reference. For each set of reads, and separately for the host and the symbiont, transcript abundance of each gene (in terms of TPM, Transcripts Per Million) was obtained with Salmon [62], using the following parameters in the quantification step: ‘--seqBias, --gcBias, --posBias, --incompatPrior 0, --recoverOrphans, --minScoreFraction 0.8, and --numGibbsSamples 110’.

### Functional annotation of the reference genomes

These proteins encoded in the macronuclear genome of *P. primaurelia* AZ9-3 were functionally annotated with eggNOG-mapper 2.1.12 [63] using the default parameters of the online tool version. The assigned protein description and GO (Gene Ontology) term annotations were taken into account (Table S2). The GO terms annotation was further refined with GOATOOLS [64]. Specifically, it was parsed to retrieve the GO hierarchical structure based on the go-basic file (https://geneontology.org/docs/download-ontology/), and, for each GO term assigned, the corresponding hierarchical level 3 and 4 were individually selected, thus producing two distinct dataframes, each containing all GO terms annotated to the respective hierarchical level. Only genes with at least one annotated GO term were considered for the transcriptome enrichment analyses. In case a gene was associated with multiple GO terms at the same hierarchical level, we treated it as if there were as many separate genes, each assigned to a single GO term.

The proteins of *Megaera* LgJac were functionally annotated using eggNOG-mapper, with the following options: ‘-m diamond --target_orthologs one2one’. For the following cluster of orthologous gene (COG) enrichment analyses of the transcriptome, only genes with at least one annotated COG were considered. In case a gene was associated with multiple COG categories, we treated it as if there were as many separate genes, each assigned to a single category.

### Gene expression analysis of *Paramecium*

In order to identify the differentially expressed genes (DEGs) between the LgJacMeg+ and LgJacMeg− strains, the TPM values of the respective three replicates were analysed using DESeq2 [65] with default parameters and the following thresholds: FDR-adjusted (False Discovery Rate) *P*-value <.05 and |log_2_FC| > 1 (Fold Change). The reference condition was set as LgJacMeg-.

Gene clustering based on log_2_FC values across replicates was performed using pheatmap [66].

Functional enrichment analysis of GO terms for up- and down-regulated genes was performed using topGO [67]. For both up- and down-regulated genes, each of the three GO categories (Biological Process—BP; Molecular Function—MF; Cellular Component—CC) and each of the two selected hierarchical levels was analysed separately. The enrichment analysis was performed using the runTest() function, applying the classical method for managing the GO graph structure and the Fisher exact test for statistical evaluation, with the *P*-values adjusted for multiple testing using the Benjamini–Hochberg (BH) method. To identify significantly enriched GO terms, an adjusted *P*-value of <.05 was applied. Data visualization was attained with ggplot2 [68]. This analysis was integrated with a detailed inspection of individual DEGs.

### Gene expression analysis of *Megaera*

The ‘poly(A)-depleted’ RNA samples sequenced for the *Megaera* RNA-Seq were expected to contain not only the targeted mRNA of this bacterium but also the non-coding RNAs (ncRNAs) of bacterium and host (besides those rRNAs effectively removed by the Ribo-Zero treatment). Therefore, to evaluate if sequencing depth was sufficient to comprehensively represent the *Megaera* transcriptome, at first, we inspected the amount of reads assigned to *Megaera*, based on the TPMs. Thus, following previous studies on bacterial symbionts [69], we performed a rarefaction analysis with vegan [70] on the reads of each replicate as well as of the three replicates merged. By this approach, it was possible to verify whether the number of expressed genes that were identified with random subsamples of each read set was comparable to those identified with the respective full set, indicative of sufficient sequencing depth. Accordingly, the combined ‘poly(A)-depleted’ reads were selected for the following analyses (see Results), and the TPMs were re-calculated for this merged read set as described above.

The *Megaera* gene expression was analysed by classifying the genes as follows. In a first classification (‘active-inactive genes’), the dataset was separated into two subgroups, namely, active genes (TPM > 0) and inactive genes (TPM = 0) (Fig. S3). A second classification (‘expression level’) was focused on the transcriptionally active genes, which were further stratified into three subgroups, namely, highly expressed genes (TPM above the 75th percentile), moderately expressed genes (TPM between the 25th and 75th percentiles), and lowly expressed genes (TPM values below the 25th percentile) (Fig. S4).

A COG category enrichment analysis was performed separately for each classification using Fisher’s exact test with BH-adjusted *P*-values, implemented in a custom R script. Similarly, for each classification, an enrichment analysis of the pangenome categories was also performed. For the ‘expression-level’ classification, the genes having TPM = 0 were excluded from the background of both enrichment analyses.

### Horizontal gene transfer analyses

All the protein sequences annotated in the *Megaera* LgJac genome were scored for being potentially the result of horizontal acquisition events. Specifically, after a blastp search on nr with the following options: -outfmt “6 std staxids” -seg no -evalue 1e-5, the resulting hits were processed using AvP [71] to obtain the Alien Index (AI), setting the ingroup as taxid = 988 779 (‘*Candidatus* Megaera polyxenophila’) and without any Excluded Genome Pool (EGP). These options allowed, respectively, to detect genes originating from non-*Megaera* taxa, and, aiming to focus on recent horizontal gene transfer (HGT) events, not to exclude any taxonomic group from the possible donors, including close relatives such as other *Rickettsiales*. Afterwards, an Alien Index threshold of >45 was applied to identify candidate HGT genes [72].

### Biotin gene synthesis phylogenetic analyses

A representative set of 254 annotated bacterial genome assemblies was prepared, aiming for a phylogenetically balanced set of *Rickettsiales* together with a selection of the best online blastp hits of the six *Megaera* LgJac biotin synthesis proteins. The LgJac sequences were then blastp-queried on each proteome, and the corresponding sequences were selected after a manual inspection. Each gene was then aligned with MAFFT [73] and trimmed with BMGE [74]. After discarding organisms bearing less than three genes, the trimmed alignments were concatenated with AMAS [75], to get a more comprehensive and informative inference. Single-gene and concatenated phylogenies were performed with IQTREE v. 2.3.4 [76], using ModelFinder [77] for model selection, with 1000 ultrafast bootstraps and 1000 SH-aLRT replicates.

## Results

### Effects of *Megaera* on host gene expression

**Figure 1:**
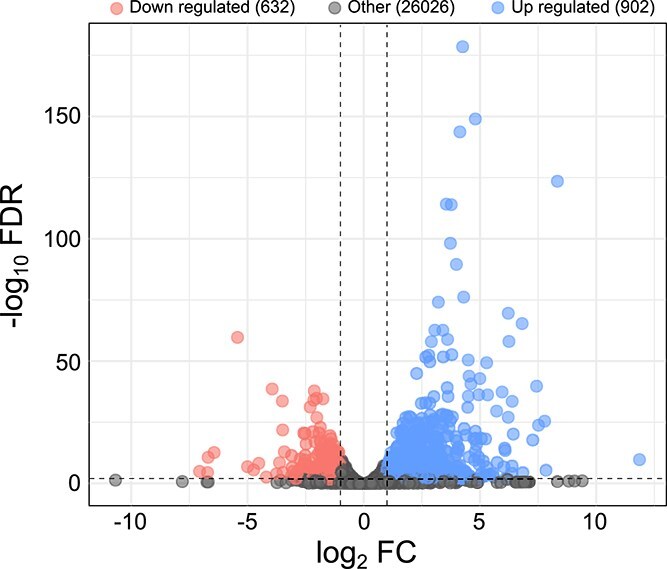
Volcano plot of the differential gene expression between *Paramecium* LgJac_Meg+ and LgJac_Meg−. The dashed horizontal and vertical lines indicate the thresholds applied for the FDR-adjusted *P*-value (.05) and for the log_2_FC (±1), respectively. Accordingly, DEGs that are up-regulated or down-regulated in LgJac_Meg+ with respect to LgJac_Meg− were selected from the other genes.

Our analyses revealed that *P. primaurelia* gene expression is appreciably influenced by the presence of *Megaera*, as numerous significant differentially expressed genes (DEGs) were identified in the presence of this symbiont, namely, 902 up-regulated and 632 down-regulated genes (Fig. 1; Table S3).

**Figure 2:**
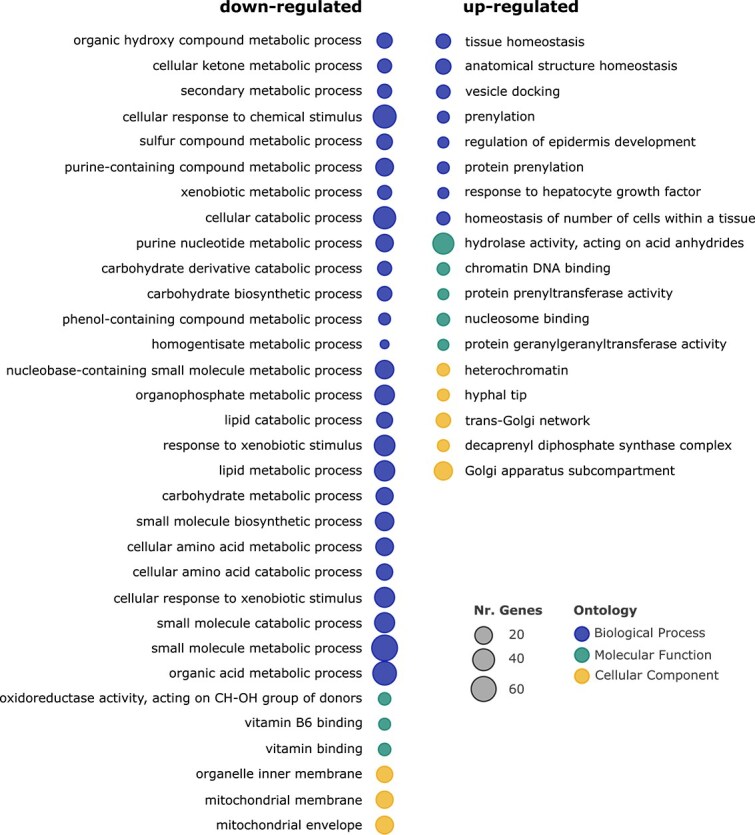
Enriched GO terms among down-regulated (left) and up-regulated (right) DEGs in LgJac_Meg+ with respect to LgJac_Meg−. GO terms are grouped according to their ontology category, namely, ‘biological process’, ‘molecular function’, and ‘cellular component’. The circle sizes are proportional to the number of DEGs assigned to the respective GO term.

Functional enrichment analysis indicated 18 enriched GO terms among up-regulated genes and 32 among down-regulated genes (Fig. 2).

Many up-regulated genes were associated with regulatory functions (Fig. 2; Table S4), suggesting a complex and fine-tuned response of *Paramecium* to the presence of *Megaera*. Up-regulated processes include transcriptional and post-transcriptional (Argonaute proteins) regulation, protein turnover (a proteasome subunit, ubiquitin-protein ligases and ubiquitin transferases, Cdc48 proteins), and signal transduction (e.g. multiple protein kinases) (Tables S3 and S5). In terms of cell compartments, several up-regulated functions localize in the Golgi apparatus and pertain to the trans-Golgi network and vesicular dynamics, in particular vesicle-docking, and secretion (e.g. SNARE proteins, phosphoinositide 3-kinases, Yip1 domain proteins). Among vesicle-trafficking genes, Rab family proteins and other GTPases, as well as their respective prenyltransferases (e.g. geranylgeranyltransferases) [78], are prominent and involved in multiple enriched GO terms (Tables S3 and S5). Several up-regulated GO terms and genes are related to nucleosome and chromatin organization and remodelling (multiple histones, histone lysine demethylases, Sir2 family histone deacetylase), being further consistent with a transcriptionally dynamic environment, also possibly indicating an overall shift towards a more condensed chromatin state. Interestingly, when inspecting in detail at the level of individual up-regulated DEGs, many of them were found to be connected to cell cycle regulation and/or division (e.g. cyclin, cyclin-kinase, anaphase-promoting complex components, Facilitates Chromatin Transcription (FACT) complex subunits, DNA replication licensing factor MCM4).

In addition, a number of up-regulated genes are linked to stress responses, including chaperones (Hsp70; HSP40/DnaJ, protein activator of Hsp90), glutathione (glutamate–cysteine ligase), and cytochrome P450 family proteins, as well as to DNA repair (Rad51).

On the other hand, most of the down-regulated GO terms are associated with metabolic processes, in particular catabolism of multiple compound classes and energy metabolism, and, consistently, with mitochondrial components (Table S4). Down-regulated pathways include general energy metabolism (Tables S3 and S6), namely Krebs cycle (e.g. 2-oxoglutarate/malate carrier, malate dehydrogenase, citrate synthases, isocitrate dehydrogenase; aconitate hydratase, fumarate hydratase and reductase, oxoglutarate dehydrogenase, succinate dehydrogenase) and oxidative phosphorylation (multiple subunits of the NADH (nicotinamide adenine dinucleotide + hydrogen)-quinone oxidoreductase and ATP - Adenosine triphosphate - synthase). Moreover, multiple compound-specific pathways are negatively affected by the presence of *Megaera*, namely, regarding carbohydrates, both glycolysis and gluconeogenesis (e.g. 6-phosphofructokinase, phosphoenolpyruvate carboxykinase, fructose-1,6-bisphosphatase, enolase, glucose-6-phosphate isomerase), as well as glycogen and glycerol degradation. Amino acid catabolism is also affected, regarding general enzymatic activities (multiple aminotransferases) and specific ones, such as degradation of glycine (cleavage system protein T), branched-chain (3-hydroxyisobutyrate dehydrogenase) or aromatic (homogentisate 1,2-dioxygenase, maleylacetoacetate isomerases, 4a-hydroxytetrahydrobiopterin dehydratase, 4-hydroxyphenylpyruvate dioxygenase) amino acids. Also, lipid catabolism is negatively affected (e.g. enoyl-CoA hydratase, acyl-CoA dehydrogenase/oxidase). Another negatively impacted function is NAD synthesis (glutamine-dependent NAD+ synthetase).

### Gene expression analysis of Megaera

We analysed the transcriptome of *Megaera* to gather insights into the mechanisms and causes for the observed gene expression shifts in the host. To this purpose, the newly assembled *Megaera* LgJac genome was used as reference (four closed replicons: chromosome: 1 724 362 bp; three plasmids: 37693, 33 040, and 5131 bp), after preliminarily confirming that the assembly quality was very high and consistent with other genomes of the same species (Table S1).

The sequencing depth of two RNA-Seq replicates was preliminarily assessed to be insufficient; therefore, to reach an adequate depth, the three replicates were merged together (Fig. S5). In the absence of a reference *Megaera* condition for a DEG analysis, we performed a global assessment of its relative gene expression levels. First, we classified genes as either expressed or nonexpressed (‘active/inactive genes’; Fig. S3). Out of 1755 annotated protein-coding genes, almost 80% (*n* = 1377) were found to be transcriptionally active. This set was strongly enriched in the COG category M (cell wall/membrane/envelope biogenesis), as well as both in the core and soft-core pangenome categories (Fig. S3; Table S7), thus indicating that highly conserved genes were preferentially expressed. On the other hand, the nonexpressed genes were complementarily enriched in the ‘cloud’ pangenome category, as well as in the COG categories L (replication, recombination, and repair) and S (function unknown) (Tables S3 and S7). Notably, L includes multiple recombinases, transposases, and DNA repair systems, which are indeed expected to be only conditionally expressed. On the other hand, several nonexpressed genes are unclassified hypothetical proteins, some of which potentially represent wrongly annotated CDSs (particularly the least conserved ones).

Next, we focused on the expressed genes, distinguishing three percentile groups (‘expression level analyses’). The highly expressed genes (over 75th percentile) were enriched in multiple functions, namely, post-translational modification, protein turnover, and chaperones (O); secondary metabolite biosynthesis, transport, and catabolism (Q); transcription (K); signal transduction mechanisms (T); and function unknown (S) (Table S7), overall indicating a metabolically active and highly regulated state. This gene set was also significantly enriched in the shell and cloud pangenome categories (Fig. S4; Table S7), suggesting that strain-specific traits may be involved in those processes. The moderately expressed gene set (25th–75th percentile) was enriched in the category D (cell cycle control, cell division, and chromosome partitioning), consistent with a stable and active replication of the bacterium (Table S7). In the lowly expressed gene subset (<25th percentile), genes related to carbohydrate transport and metabolism (category G) were more represented, which may be indicative of a stable and relatively measured need for such functions within the host environment, also considering that many of those genes encode for membrane transporters. Conversely, no significant enrichment was detected in pangenome categories among the moderately and lowly expressed genes (Table S7).

Thus, we moved to an in-depth investigation at the single gene level. As expected for a *Rickettsiales* bacterium, *Megaera* LgJac expresses typical core (or soft core) components of secretion systems (Sec, Tat, and T4SS). Interestingly, an F-type conjugative pilus, moderately shared with other *Megaera* (mostly soft core/cloud), was also expressed. Among putative effectors, three ankyrin repeat proteins (two in the highly expressed genes) and five tetratricopeptide repeat proteins (one among the highly expressed genes), as well as one pentapeptide repeat protein are expressed, all of which are only poorly conserved (cloud). Besides, *Megaera* expresses several *Rickettsia*-like factors involved in host cell invasion, including Sca2, Sca4, patatin-like phospholipase and phospholipase D (core) [79, 80]. Interestingly, it also expresses multiple toxins and antitoxins, in particular four possible pairs among highly expressed genes. Most of those are not shared with other *Megaera* (cloud) and are likely involved in plasmid or other kinds of regulation but could also take part in host interactions [37]. *Megaera* also expresses five non-ribosomal peptide synthesis domain proteins (including thioesterase and gramicidin dehydrogenase-like), previously suggested to be possibly implied in defensive mutualism [30].

*Megaera* expresses several metabolic pathways that are down-regulated in *Paramecium* in its presence, in particular energy metabolism (pyruvate dehydrogenase; Krebs cycle; and oxidative phosphorylation complexes I, III, IV, and V, as well as the alternative bd oxidase). It also expresses five tlc nucleotide translocases, which may allow for the exchange of energy-carrying nucleotides with the host. Moreover, it bears transporters and enzymes (e.g. NAD-specific glutamate dehydrogenase, threonine dehydratase) enabling scavenging small compounds from the host and fuelling the Krebs cycle directly by its intermediates. When considering potentially host-supportive actions, *Megaera* is actively performing the synthesis of multiple vitamins and cofactors, namely, ubiquinone, lipoate, heme, folate, CoA, and biotin.

### *Megaera* horizontally acquired a putatively host-supportive biotin synthesis operon

Subsequently, we focused on investigating the genome organization and evolutionary origin of the *Megaera* LgJac biotin synthesis genes. Indeed, while not within the highest expressed fraction (Table S7), these genes are almost absent in the other *Megaera* (shell)*.* Conversely, the biosynthetic pathways of other vitamins and the biotin transporter *bioY* are all highly conserved (core or soft core), hinting at a possible HGT of biotin synthesis genes. Moreover, the eggNOG annotation of *P. primaurelia* indicates that this ciliate is devoid of such a pathway.

**Figure 3:**
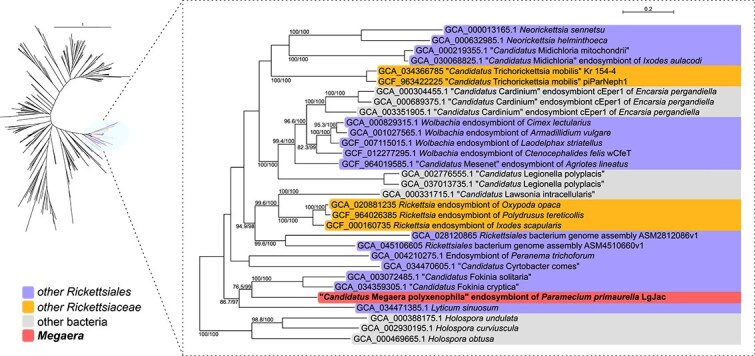
Maximum likelihood unlabelled tree of the concatenated biotin synthesis genes inferred with the Q.pfam + I + G4 model (left). The shaded branches, corresponding to the BOOM clade, are shown labelled in the dashed enlarged version on the right. On each branch, support values by SH-aLRT with 1000 replicates and by 1000 ultrafast bootstraps are reported (bootstrap values below 90 were omitted). The newly sequenced *Megaera* Lg_Jac is evidenced in bold. The branch colour (left) and background colour (right) indicate the organism lineages, namely, *Megaera*, other *Rickettsiaceae*, other *Rickettsiales*, and other bacteria. The scale bars stand for the estimated proportional sequence divergence. The full tree is shown in Fig. S6.

The six biotin synthesis genes are all in close proximity in the *Megaera* LgJac genome, namely, five of them consecutive on the same strand (*bioB* is consecutive but on the opposite strand), thus suggesting that they might constitute an operon. The same gene arrangement was repeatedly identified in bacterial symbionts of insects and other arthropods, with several inferred HGT events and a role in nutritional mutualism [81–83], and accordingly termed BOOM (‘Biotin synthesis Operon of Obligate intracellular Microbes’) [84]. Interestingly, the six biotin synthesis genes of *Megaera* LgJac had very high Alien Index scores (all >270, 3/6 with the top highest value; Table S8), which is further indicative of HGT. This was confirmed by phylogenies of every single gene and on the six concatenated genes, which were all consistent in placing the *Megaera* LgJac sequences within the same clade of BOOM sequences (hereafter, ‘BOOM clade’) with high support (ultrafast bootstrap ≥90% in all single proteins besides BioD, and 100% in the concatenated alignment; Figs 3, S6, and  S7). This clade also encompasses numerous other *Rickettsiales* symbionts of ciliates and other protists (e.g. ‘*Candidatus* Fokinia spp.’, *Lyticum sinuosum*, the symbiont of *Peranema trichophorum*, ‘*Candidatus* Trichorickettsia mobilis’).

Within the BOOM clade, the relationships between *Rickettsiales* sequences and those from other bacteria, as well as among *Rickettsiales*, are quite interwoven and not consistent with organismal phylogeny (Figs 3 and S6). In particular, in the concatenated alignment, the *Megaera* LgJac sequences are not proximate with their closest relatives among the family *Rickettsiaceae* (*Rickettsia* and ‘*Candidatus* Trichorickettsia’) but rather nested with very high support (bootstrap 97%) into a clade of ‘*Candidatus* Midichloriaceae’ symbionts of *Paramecium*, namely, ‘*Candidatus* Fokinia spp.’ and *L. sinuosum*. Single gene phylogenies are consistent and, remarkably, indicate that the only other biotin synthesis enzyme found in a *M. polyxenophila*, namely, a plasmid-encoded BioA from strain PCS, is very distant from LgJac and other *Rickettsiales* sequences in general (Fig. S7).

On the other hand, other *Rickettsiales* sequences, including those of symbionts of ciliates such as ‘*Candidatus* Grellia numerosa’, form clades that are fully apart from the BOOM clade (Figs 3, S6, and  S7).

Collectively, these results highly support that this operon was horizontally acquired by *Megaera* LgJac from other symbionts of *Paramecium*, probably quite recently, after the divergence from other *Megaera*. Consistent with this scenario, multiple genes linked to mobile elements (transposases, phages, plasmid toxins), which may have acted as carriers for the biotin synthesis genes, were also retrieved among the HGT candidates with high Alien Index scores (Table S8).

## Discussion

Here, we investigated the effect of the *Rickettsiales* bacterium *Megaera* on its host *Paramecium* strain LgJac_Meg+, leveraging a combination of dual RNA-Seq and comparative genomics to understand the functional features of this interaction from an evolutionary perspective. While protists are prominent and ancestral hosts for *Rickettsiales* [11], to our best knowledge, this is the first study exploring a *Rickettsiales*–protist association through transcriptomics.

Overall, we identified 1534 *Paramecium* DEGs in response to the presence of *Megaera*. In *Paramecium*, a similar number of DEGs was detected in response to *Caedibacter taeniospiralis* (*Fastidiosibacteraceae*, *Gammaproteobacteria*) [48], while a markedly higher one was found in response to certain environmental changes [85], further suggesting that the effects of persistent bacterial symbionts on ciliates are relatively contained and fine-tuned. Nevertheless, the functions involved indicate quite substantial effects linked to the presence of *Megaera*, considering in particular the several up-regulated regulatory functions, including regulation over gene expression. The up-regulation of genes involved in chromatin condensation dynamics, DNA replication, and cell division, as well as in vesicular dynamics and Golgi-related processes, seems consistent with the previously observed higher growth rate of LgJac_Meg+ line as compared to LgJac_Meg− [25]. On the other hand, the prominent down-regulated functions are associated with catabolism of carbohydrates, amino acids, and lipids, as well as energy metabolism, namely, the Krebs cycle and oxidative phosphorylation. This seems unexpected at a first glance, considering that, as typical among *Rickettsiales* [2, 38], genomics would predict that *Megaera* relies heavily on host metabolism for energy, being supplied of intermediates for its energy metabolism as well as potentially importing ATP directly, thanks to its five tlc nucleotide translocases. However, this apparent paradox can be solved if one considers that *Megaera* is equipped with its own Krebs cycle and oxidative phosphorylation, with the produced ATP that could also become available for *Paramecium,* assuming the tlc transporters are acting in reverse [86]. This might effectively compensate the reduced expression of the corresponding host pathways, at least upon certain conditions. Accordingly, the higher *Paramecium* growth could be due to the fact that such surrogate of host metabolic functions by the symbiont might result in a more efficient exploitation of available resources, possibly due to compartmentalized functions [87], somehow analogous to cell organelles [88]. An alternative and nonmutually exclusive explanation is that such differences in growth observed by Pasqualetti and co-authors could be at least partly due to some long-term effect of the antibiotic treatment and/or subcloning [25]. In any case, the hypothesized involvement of *Megaera* would imply that this symbiont can behave as a well-integrated mutualist. This would occur provided that a sufficient food supply compensates for the overall energetic cost that *Megaera* is expected to burden the host with, given that this bacterium was not shown to broaden the range of molecules usable as food sources. Along the same line of thought, the possible implication of biotin seems noteworthy. While *Paramecium* is unable to produce this vitamin, *Megaera* expresses the biotin synthesis genes and a biotin transporter, so it could provide it to its host, further contributing to a mutualistic effect. This hypothesized biotin provision is reminiscent of the role of multiple nutritional symbionts of arthropods [81, 83, 84]. However, the bacterivorous diet of *Paramecium* and other ciliates is more varied [89], entailing that both the aposymbiotic LgJac_Meg− and at least partly the symbiotic LgJac_Meg+ may exploit biotin produced by food bacteria. Overall, this suggests a quantitative and facultative benefit provided by *Megaera*, with possible competitive advantages, which is fully consistent with experimental data, indicating a stable but nonobligate association [25].

Interestingly, *C. taeniospiralis* causes almost opposite effects on the gene expression of *Paramecium* (increased catabolism, reduced expression of proteins involved in the cell cycle) [48] and, consistently, has no tlc transporters or biotin synthesis or transporters [49]. On the other hand, ‘*Preeria caryophila*’ (*Holosporaceae*, *Alphaproteobacteria*) was reported to have positive effects on the host growth rate, reminiscent of *Megaera* [90]. While no ‘*Preeria*’ genome is currently publicly available, its close relatives, *Holospora* spp., have neither the Krebs cycle nor oxidative phosphorylation but do have biotin synthesis [91, 92], thus hinting at a possible similar host-supportive mechanism.

From an evolutionary perspective, all the other 12 analysed *M. polyxenophila* genomes do not encode for biotin synthesis, while the Alien Index and gene phylogenies robustly indicate that *Megaera* LgJac acquired quite recently the corresponding BOOM operon through HGT from other symbionts of *Paramecium*, such as ‘*Candidatus* Fokinia spp.’ or *L. sinuosum* (Figs 3, S6, and  S7). In general, multiple events of transfer of BOOM have likely occurred between *Rickettsiales* and other bacterial symbionts, hosted by arthropods and protists. Interestingly, the presence of conjugal transfer machinery in many of those symbionts [2, 24, 93, 94], including LgJac and other *M. polyxenophila*, as well as of multiple mobile elements such as plasmids, transposases, and phages, suggests possible mechanisms that could support recurring exchanges of genetic material among them and/or with other bacteria. These results reinforce the notion that HGT events might be relevant for the evolution of host interactions of protist-associated *Rickettsiales* [18]. For what concerns biotin synthesis genes, these events are probably rather common and often followed by successive losses, due to changing evolutionary pressures according to host organisms and environmental conditions. This is also supported by the fact that just a single evolutionarily unrelated biotin synthesis gene (BioA) was found in only another *M. polyxenophila* strain and that another symbiont of *Paramecium* (‘*Candidatus* Sarmatiella mevalonica’) has a single distinct gene (BioB), which, in addition, is closely related to that of *Megaera* LgJac (Fig. S7).

*Megaera* also expresses nonribosomal protein synthases, some of which are expressed at very high levels, which have been previously suggested to be implied in defensive mutualisms [30]. In the context of laboratory cultivation, the role of these proteins is unclear.

Other than possible host-supportive functions, *Megaera* also bears and expresses genes relatable to exploitative actions on the host, such as homologs of *Rickettsia* host cell invasion proteins [79, 80] and a number of toxin–antitoxin modules, many of which are at a high level. Indeed, besides regulation of plasmid copy number and other bacterial cell physiological traits [95], for the latter, a role in the manipulation of host cells was suggested, in particular for tightening the association by preventing symbiont loss through addiction mechanisms [37]. The presence of secretion systems and effectors is also suggestive of an active modulation of the host cell by *Megaera*, possibly including a direct mediation of the observed gene expression variations. Among *Paramecium* up-regulated DEGs, we also found stress response chaperones such as Hsp70, which was previously found to be over-expressed by *Paramecium* in the presence of *C. taeniospiralis* and *Holospora elegans*, enabling the ciliate to better cope with heat shock [48, 96]. This finding is further consistent with a somewhat ambivalent role of *Megaera* for its host.

Overall, our results suggest multifaceted interactions between *Megaera* and its hosts, with possible physiological flexibility according to host–symbiont combinations and environmental factors, and potentially with adaptive evolutionary changes in gene content. Under this scenario, individual genes would exert multiple roles, e.g. tlc and bioY transporters may allow metabolite scavenging or alternatively be involved in their provision to the host. This suggests an intriguing parallelism with the well-studied case of *Wolbachia,* which is also a widely diffused *Rickettsiales* bacterium with a broad host range [7]. *Wolbachia* is able to addict its hosts through reproductive manipulation, but certain lineages have evolved into obligate mutualists, including biotin-providing ones, leading to its definition as a ‘master manipulator of invertebrates’ [97]. This evolutionary parallelism is also strengthened when considering other protist-associated *Rickettsiales.* Indeed, early studies showed that *Lyticum* (whose biotin operon is a close relative of the LgJac *Megaera* one) may behave both as a vitamin provider (folate) [98] and as a host killer [99, 100], which is an instance of an addictive manipulator [37].

Taking all the above into account, it is suggested that *Rickettsiales* in general (and potentially other lineages of the ‘professional symbionts’, such as *Holosporineae*, *Legionellales*, and *Chlamydiota* [101]) engage in complex and flexible interactions with protists. These bacteria, while having a primary capability to invade and exploit host cells, fulfil a fine-tuned sum of multiple actions, with the possibility to conditionally switch or evolve towards overall more positive interaction modes, including vitamin provision. This flexibility could be at the base of their wide diffusion and success in diverse hosts, in particular for clades such as *Megaera*, in which phylogenetically proximate bacteria are associated with a broad host range [27, 30].

Further studies are needed to functionally characterize the *Megaera*–*Paramecium* and other such interactions, in particular aiming to correlate the effect of physiological and environmental changes with potential molecular determinants, as well as accounting for a broader range of phenotypic effects, including behavioural ones. After seminal works initiated decades ago (e.g. [96–98]), thanks to the power of modern laboratory techniques and *in silico* approaches, a novel phase of experimental work on these symbiotic associations seems now possible, with a great potential for shedding light on their functional and ecological features, as well as on their evolution.

## Supplementary Material

Supplementary_material_ycag079

## Data Availability

The genomic sequencing reads and assembled *Megaera* LgJac genome and the transcriptome reads have been deposited under the NCBI Bioproject PRJNA1297651.
