# Supplementary material for: Facultative mutualism between Paramecium and the intracellular Rickettsiales bacterium Megaera mediated by a horizontally acquired biotin operon
Source: ISME Commun. 2026 Mar 27;6(1):ycag079. doi: 10.1093/ismeco/ycag079 (PMC13134042; doi:10.1093/ismeco/ycag079)
Supplement: Supplementary_material_ycag079 [file supplementary_material_ycag079.zip › Figure S3_active-inactive_updated2026.pdf]

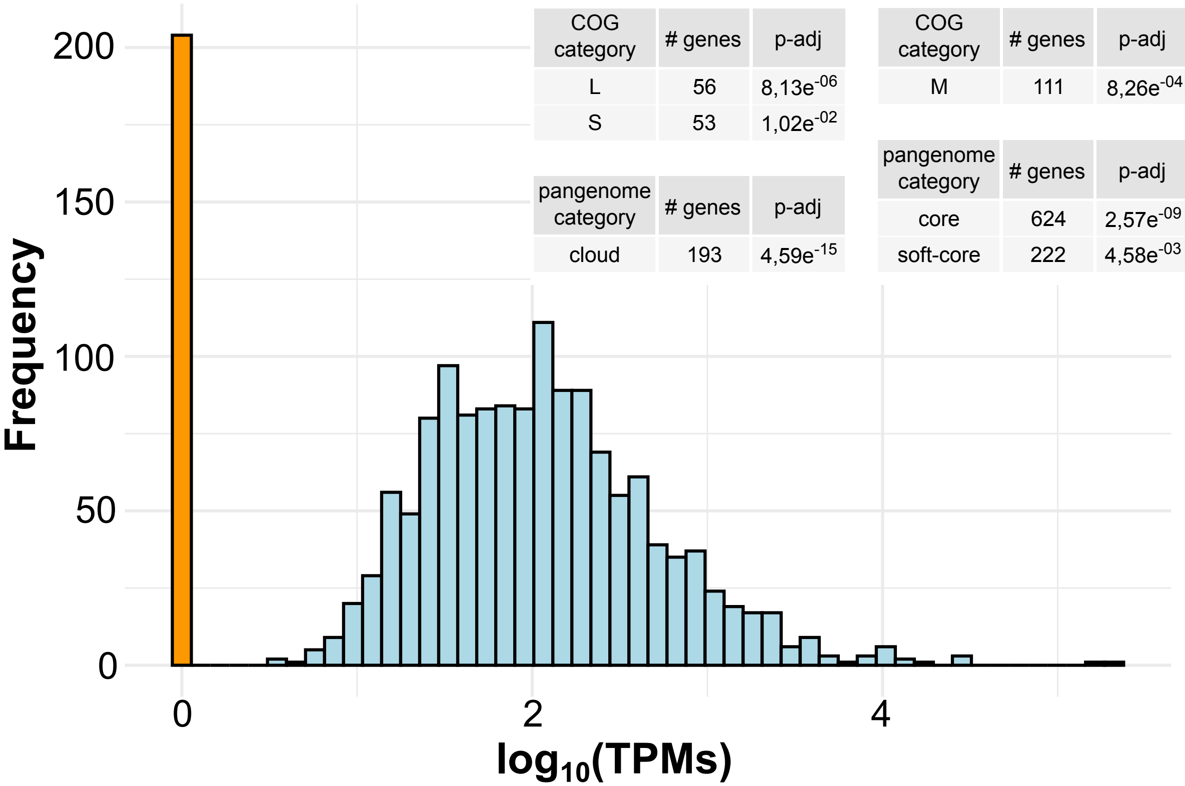

Inactive genes (TPMs = 0)

COG category	# genes	p-adj
L	56	$8,13e^{-06}$
S	53	$1,02e^{-02}$

pangenome category	# genes	p-adj
cloud	193	$4,59e^{-15}$

Active genes (TPMs > 0)

COG category	# genes	p-adj
M	111	$8,26e^{-04}$

pangenome category	# genes	p-adj
core	624	$2,57e^{-09}$
soft-core	222	$4,58e^{-03}$
