# Supplementary material for: Facultative mutualism between Paramecium and the intracellular Rickettsiales bacterium Megaera mediated by a horizontally acquired biotin operon
Source: ISME Commun. 2026 Mar 27;6(1):ycag079. doi: 10.1093/ismeco/ycag079 (PMC13134042; doi:10.1093/ismeco/ycag079)
Supplement: Supplementary_material_ycag079 [file supplementary_material_ycag079.zip › Figure S4_expression_level_updated2026.pdf]

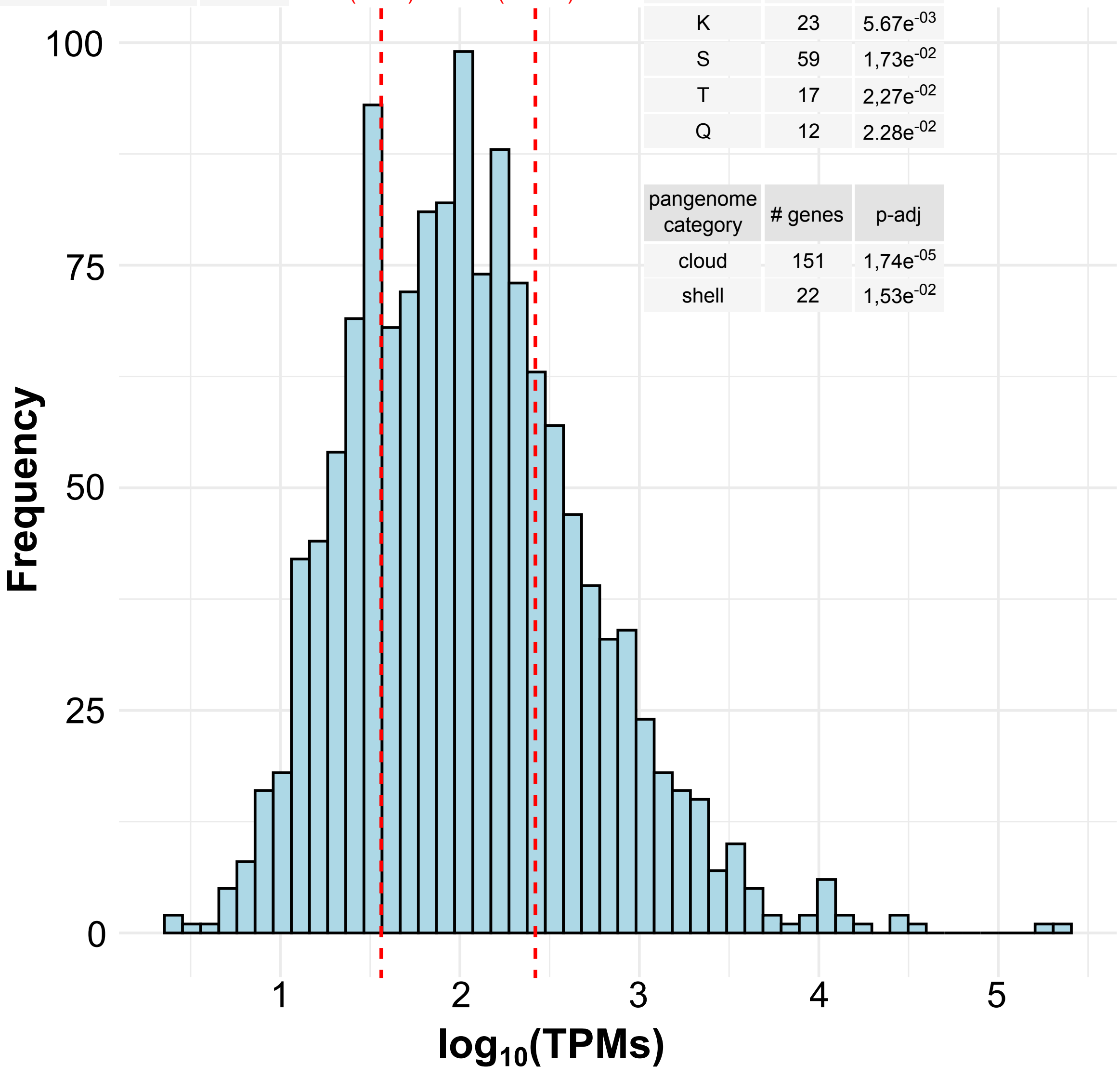

Lowly expressed genes

COG category	# genes	p-adj
G	22	$4{,}49e^{-02}$

Moderately expressed genes

COG category	# genes	p-adj
D	22	$3{,}36e^{-02}$

Highly expressed genes

COG category	# genes	p-adj
O	23	$5{,}67e^{-03}$
K	23	$5.67e^{-03}$
S	59	$1{,}73e^{-02}$
T	17	$2{,}27e^{-02}$
Q	12	$2.28e^{-02}$

pangenome category	# genes	p-adj
cloud	151	$1{,}74e^{-05}$
shell	22	$1{,}53e^{-02}$
