# Supplementary material for: Facultative mutualism between Paramecium and the intracellular Rickettsiales bacterium Megaera mediated by a horizontally acquired biotin operon
Source: ISME Commun. 2026 Mar 27;6(1):ycag079. doi: 10.1093/ismeco/ycag079 (PMC13134042; doi:10.1093/ismeco/ycag079)
Supplement: Supplementary_material_ycag079 [file supplementary_material_ycag079.zip › Legend_supplementary.pdf]

Legends of the Supplementary materials

Figure S1: Blobology plot of the preliminary assembly of the Lg_Jac sample. Contigs are shown according to their GC content and log 10 of sequencing coverage, and coloured according to the respective best megablast hit. Only contigs with length higher than or equal to 1000 bp are shown for viewers' clarity.

Figure S2: Diagram of the workflow of the transcriptomics experiment

Figure S3: Barplot showing the TPM frequency in the *Megaera* transcriptome. Genes are split according to the "active-inactive genes" analysis, namely into inactive genes (TPM=0; orange), and active genes (TPM>0; light blue). The Table inset show the results of the enrichment analyses for COG and pangenome categories in each of the two groups. For viewers' clarity, only significant categories are displayed.

Figure S4: Barplot showing the TPM frequency in the *Megaera* transcriptome among the active genes (TPM>0). Genes are split according to the "expression level" analysis, namely into "highly expressed" (TPM above the 75th percentile), "moderately expressed" (TPM between the 25th and 75th percentiles), and "lowly expressed" genes (TPM values below the 25th percentile). The Table inset show the results of the enrichment analyses for COG and pangenome categories in each of the three groups. For viewers' clarity, only significant categories are displayed.

Figure S5: Saturation curves of the expressed genes detected with the total reads of each of the three replicates and their merge, and with their progressive subsamples.

Figure S6: Full phylogenetic tree of the concatenated biotin synthesis genes, inferred with the Q.pfam+I+G4, as predicted with ModelFinder. An arbitrary root was put for visualisation purposes only. The newly obtained sequences of *Megaera* LgJac is evidenced in bold. The coloured backgrounds indicate sequences of *Megaera* (red), other *Rickettsiaceae* (orange), and other *Rickettsiales* (purple). On each branch, support values by SH-aLRT with 1000 replicates and by 1000 ultrafast bootstraps are reported. The tree scale stands for estimated proportional sequence divergence.

Figure S7: Phylogenetic trees of each the biotin synthesis genes. Arbitrary root were put for visualisation purposes only. The newly obtained sequences of *Megaera* LgJac are evidenced in bold. The coloured backgrounds indicate sequences of *Megaera* (red), other *Rickettsiaceae* (orange), and other *Rickettsiales* (purple). On each branch, support values by SH-aLRT with 1000 replicates and by 1000 ultrafast bootstraps are reported. Each tree was inferred with a substitution model predicted with ModelFinder (BioC, BioH, BioF, and BioD: Q.pfam+I+G4; BioA and BioB: LG+G4+I). The tree scales stand for estimated proportional sequence divergence.

Table S1: Summary of the BUSCO completeness analysis of the Megaera polyxenophila genomes. Samples discarded according to the selected cutoffs (>80% complete and single copy reference orthologs; <2% duplicated reference orthologs) are marked in yellow.

Table S2: eggNOG annotation of the *Paramecium primaurelia* genome

Table S3: Full lists of up-regulated and down-regulated *Paramecium* DEGs

Table S4: Significantly enriched GO terms among up-regulated and down-regulated *Paramecium* DEGs, and the respective gene IDs. Background colours indicate the respective ontology category (BP: biological process; CC: cellular component; MF: molecular function).

Table S5: List of up-regulated DEGs assigned enriched GO terms, arranged according to the ontology category, which also determines the background colours (BP: biological process; CC: cellular component; MF: molecular function).

Table S6: List of down-regulated DEGs assigned enriched GO terms, arranged according to the ontology category, which also determines the background colours (BP: biological process; CC: cellular component; MF: molecular function).

Table S7: For each annotated *Megaera* LgJac gene, the respective TPM, the corresponding "active-inactive" and "expression level" classification, eggNOG annotation (including COG category) and pangenome category are shown.

Table S8: Selected candidate HGT genes of *Megaera* LgJac, ordered by AI values, with a threshold of 45. The HGT parameters predicted by AvP are shown, together with the overall best hit on NCBI nr, and, when available, on other Megaera sequences, as well as the eggNOG annotation. The six biotin synthesis genes are highlighted in green
