# Supplementary figures and images for: Facultative mutualism between Paramecium and the intracellular Rickettsiales bacterium Megaera mediated by a horizontally acquired biotin operon
Source: ISME Commun. 2026 Mar 27;6(1):ycag079. doi: 10.1093/ismeco/ycag079 (PMC13134042; doi:10.1093/ismeco/ycag079)

## Cell lines

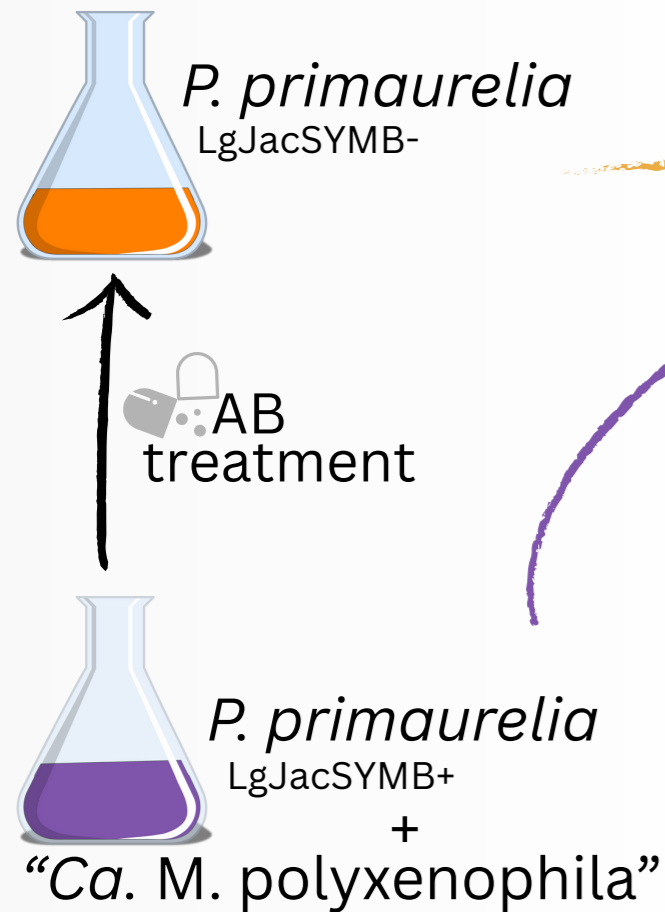

## Sample preparation

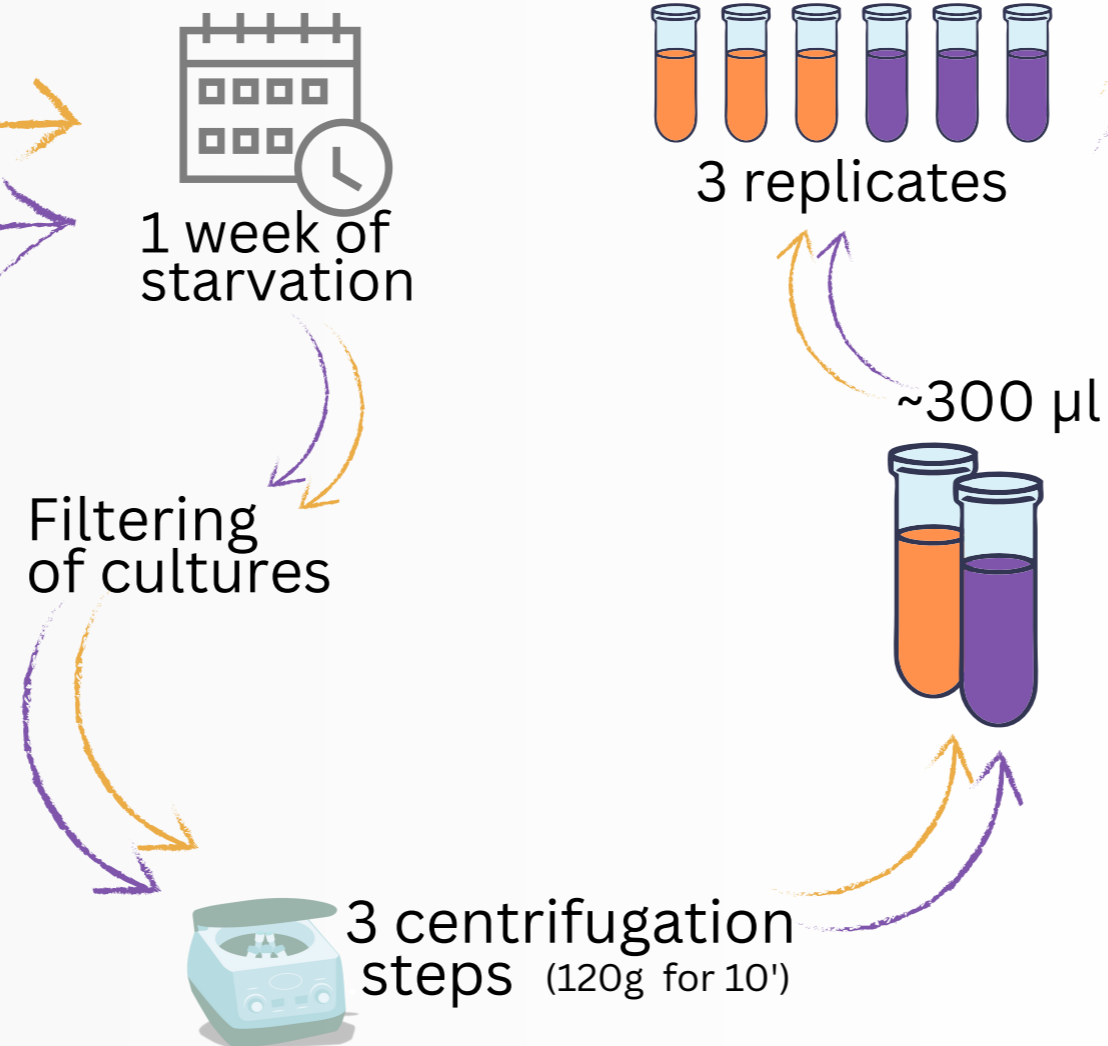

## RNA extraction and processing

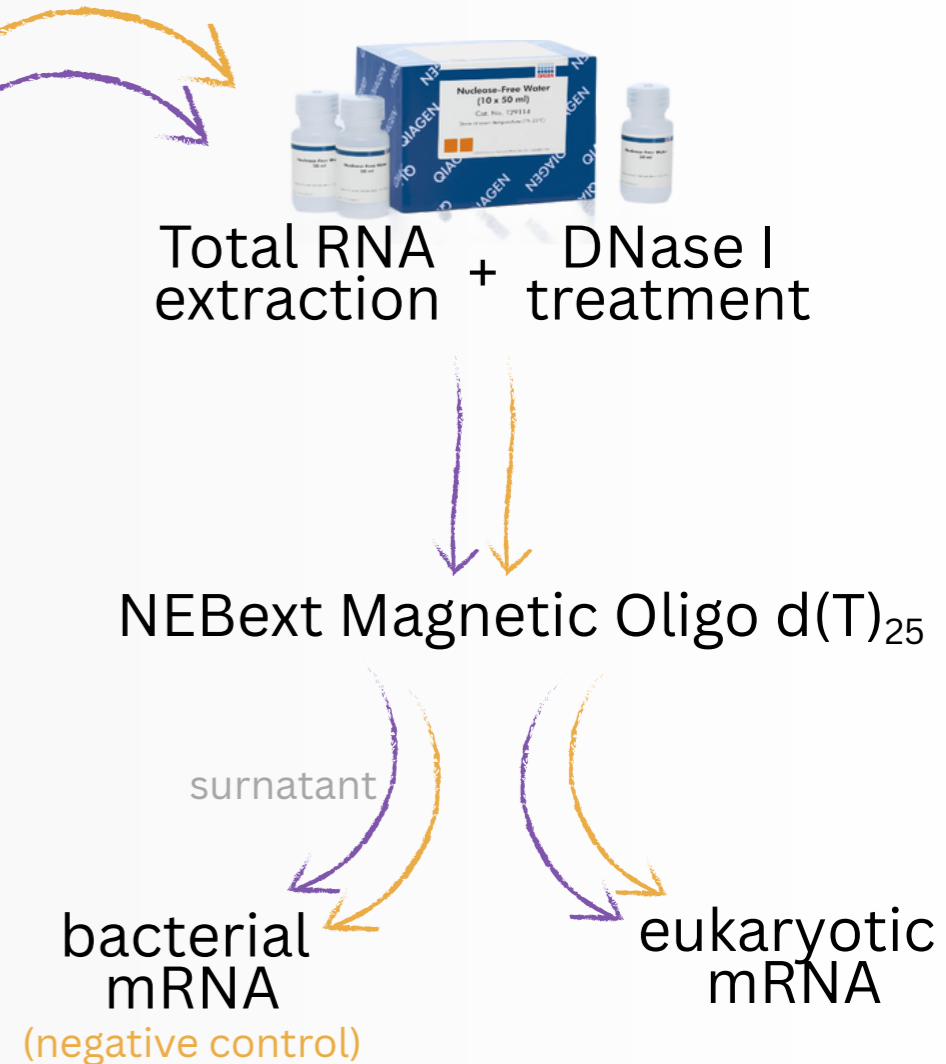

Supplement: Supplementary_material_ycag079 [file supplementary_material_ycag079.zip › Figure S2_workflow.pdf]

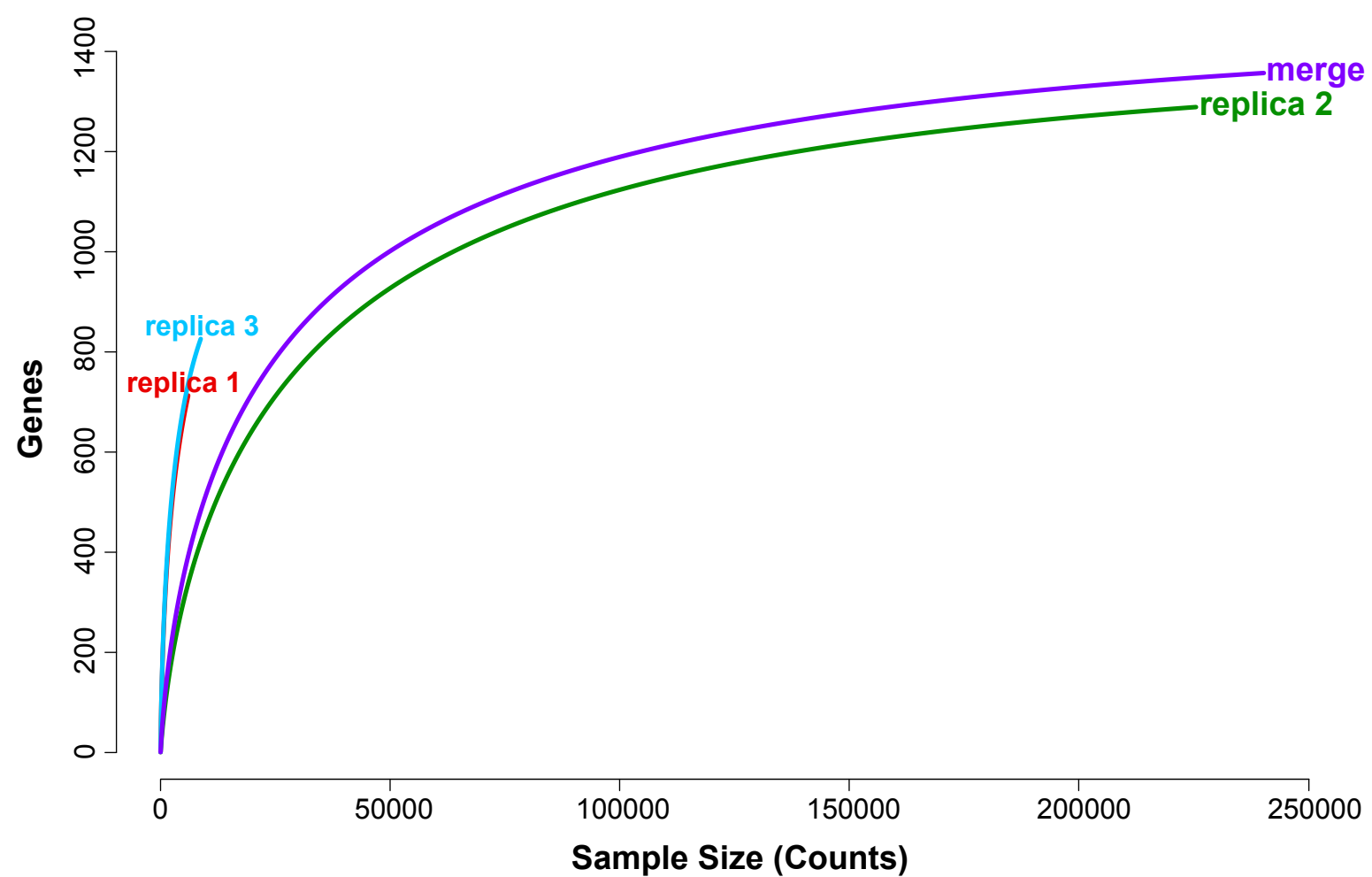

Supplement: Supplementary_material_ycag079 [file supplementary_material_ycag079.zip › Figure S5_saturation_curves_updated2026.pdf]

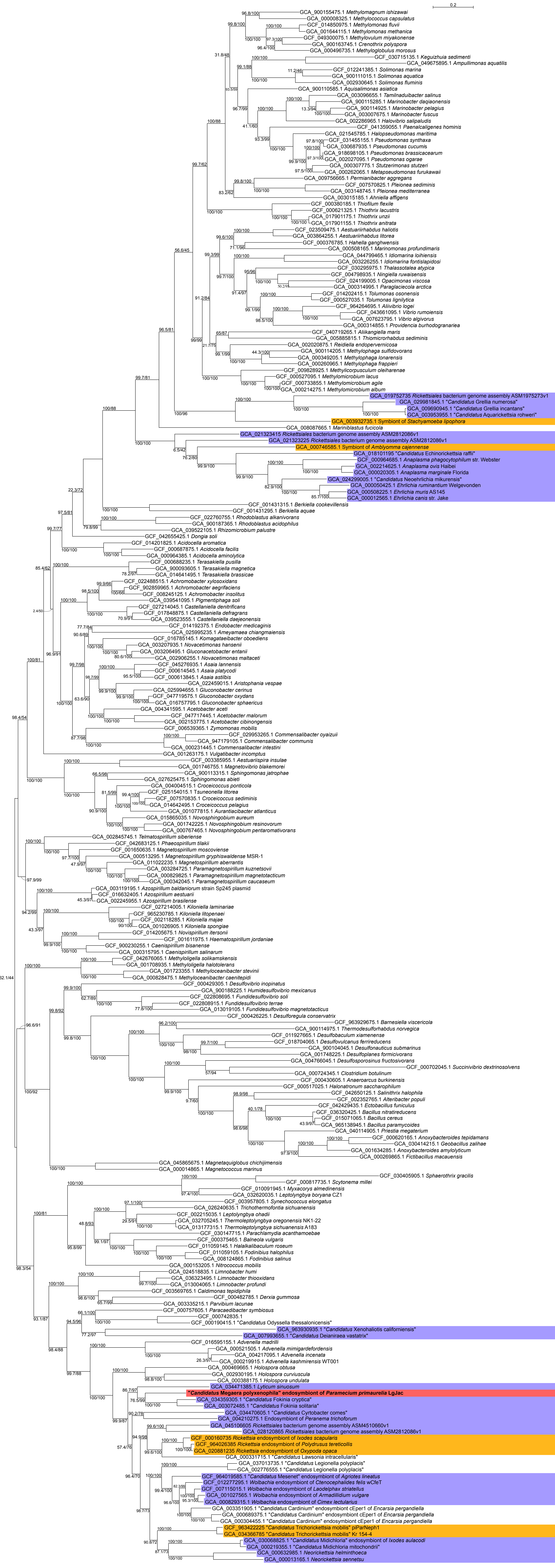

Supplement: Supplementary_material_ycag079 [file supplementary_material_ycag079.zip › Figure S6_concatenated_tree_updated.pdf]

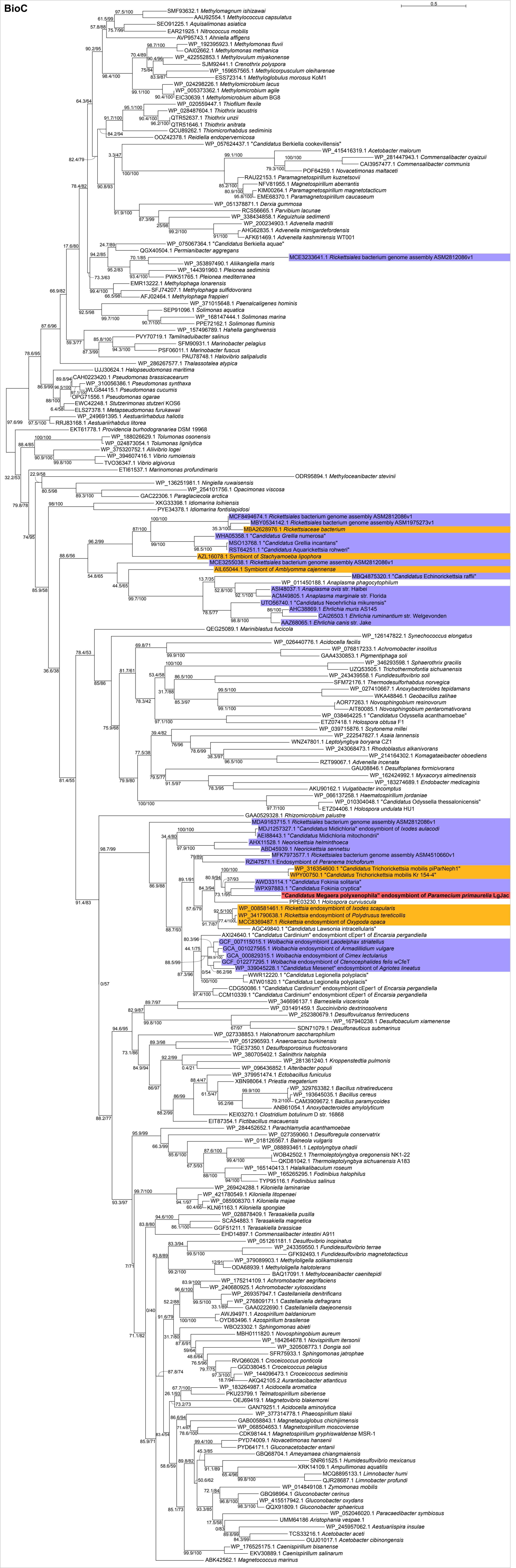

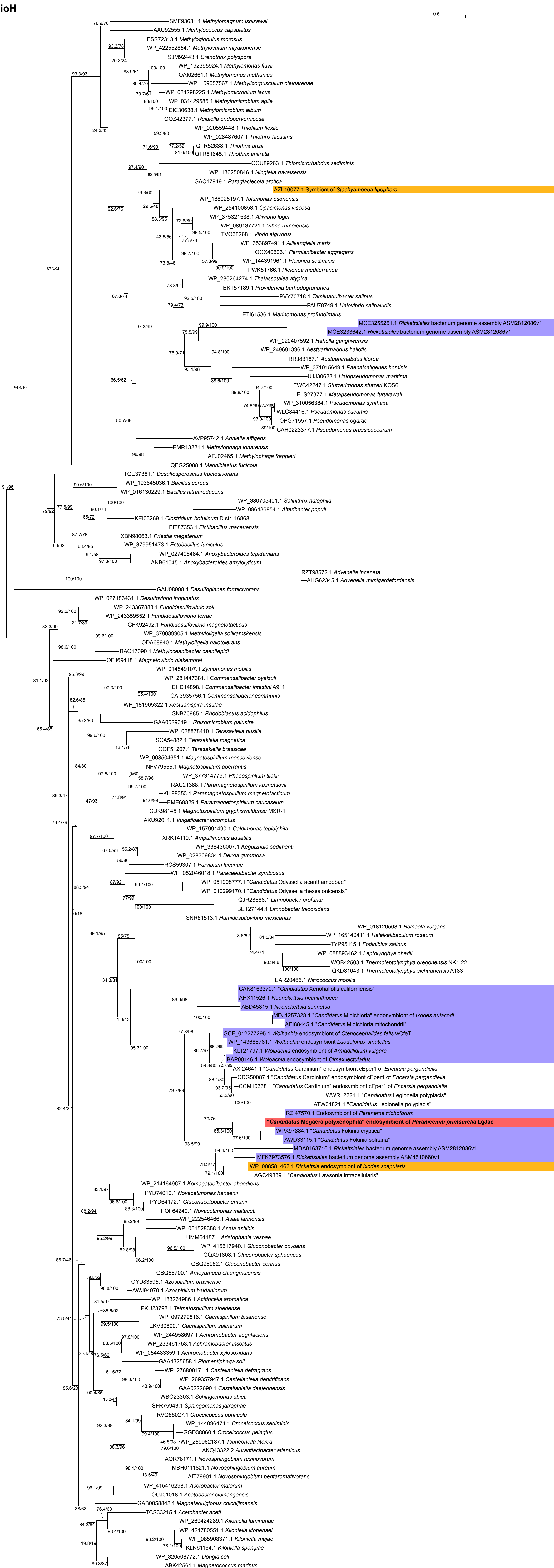

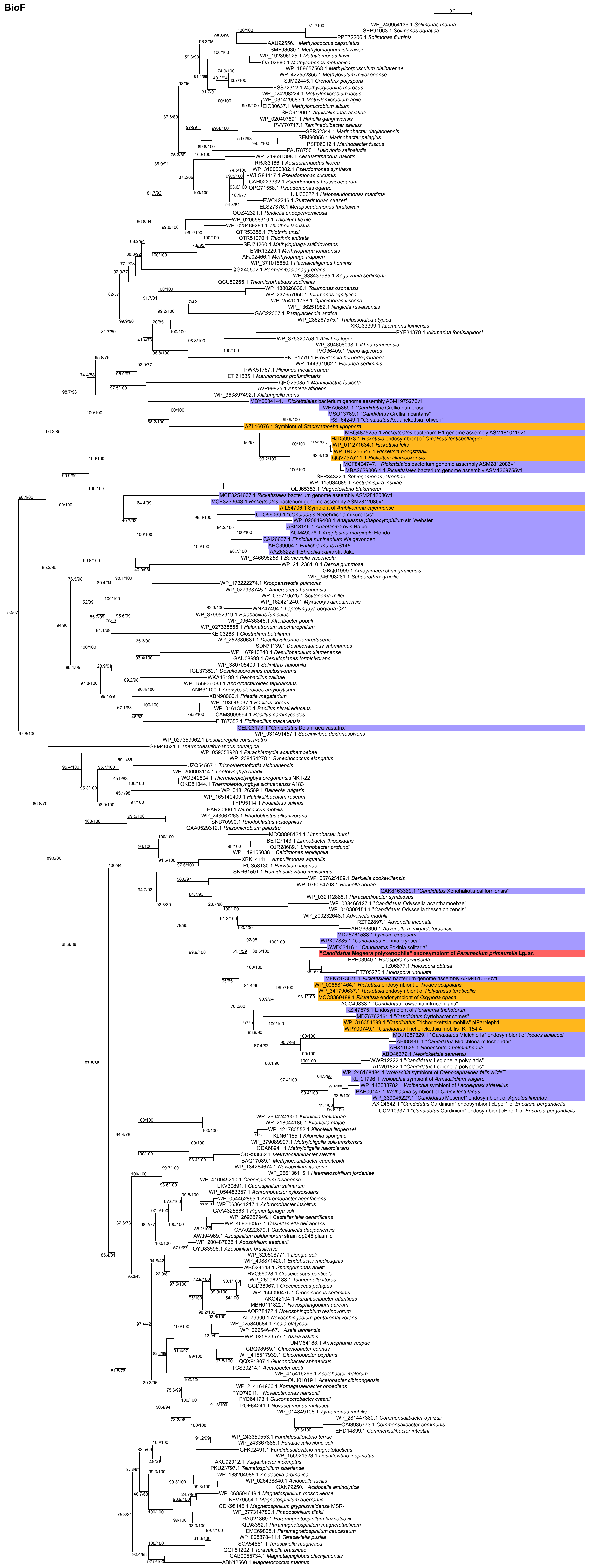

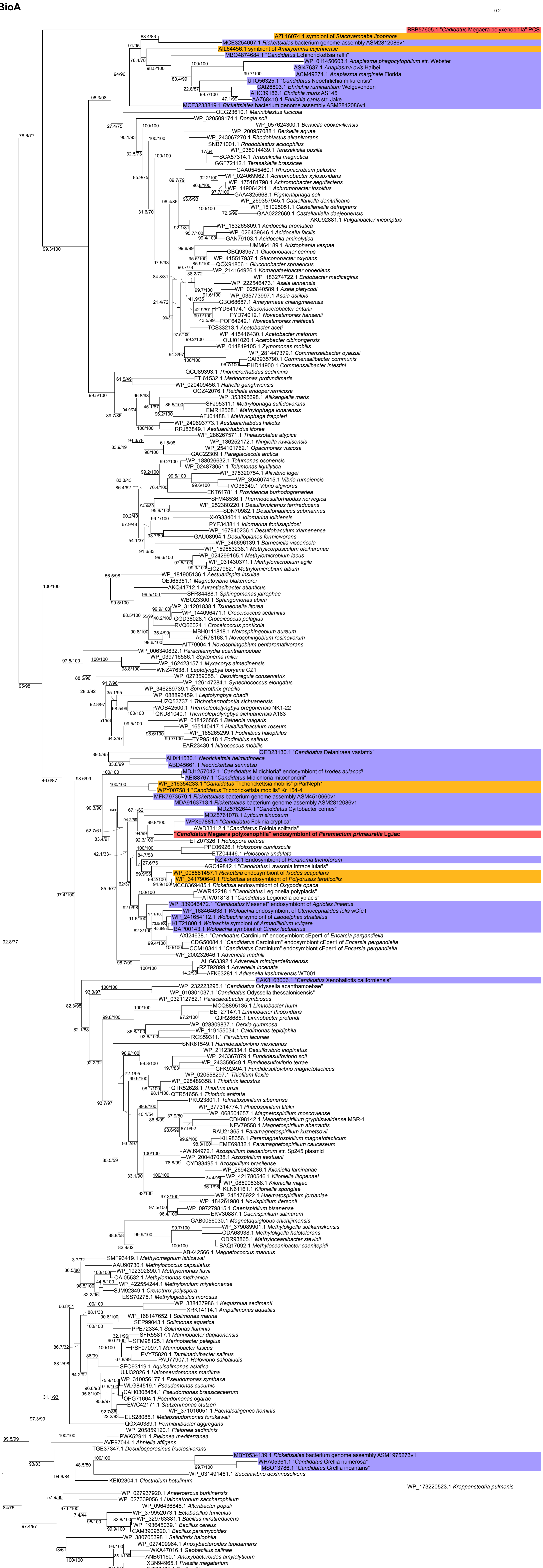

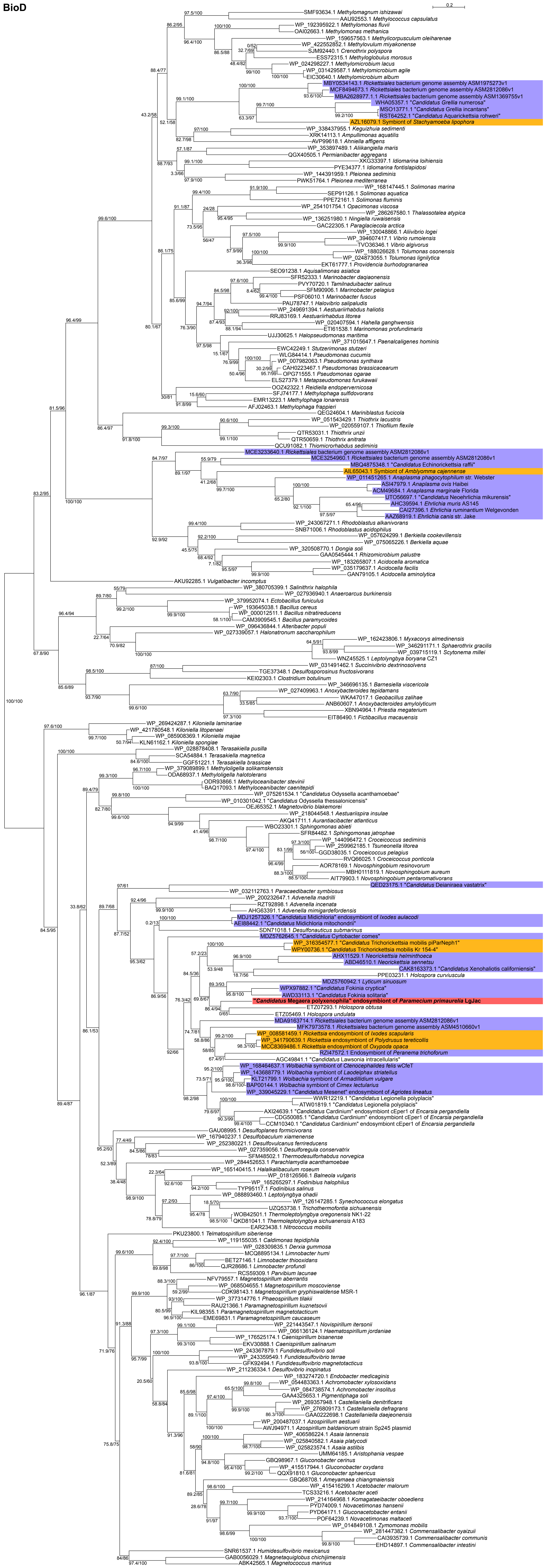

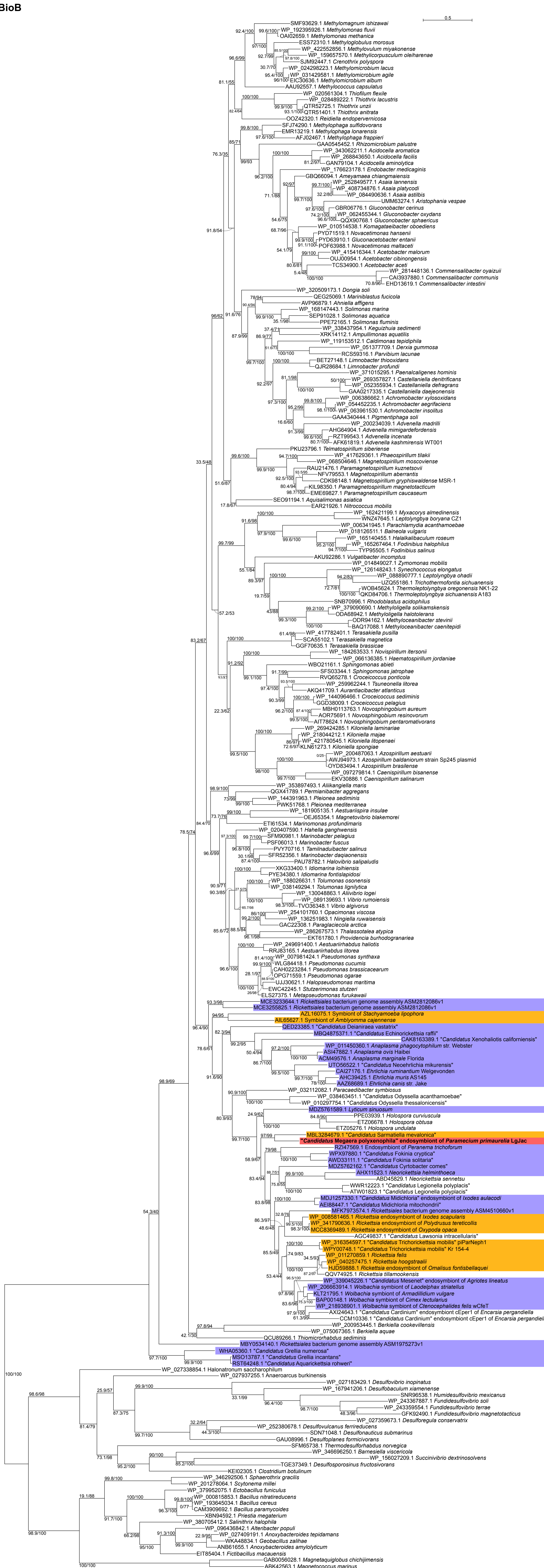

Supplement: Supplementary_material_ycag079 [file supplementary_material_ycag079.zip › Figure_S7_single_genes_trees_updated.pdf]

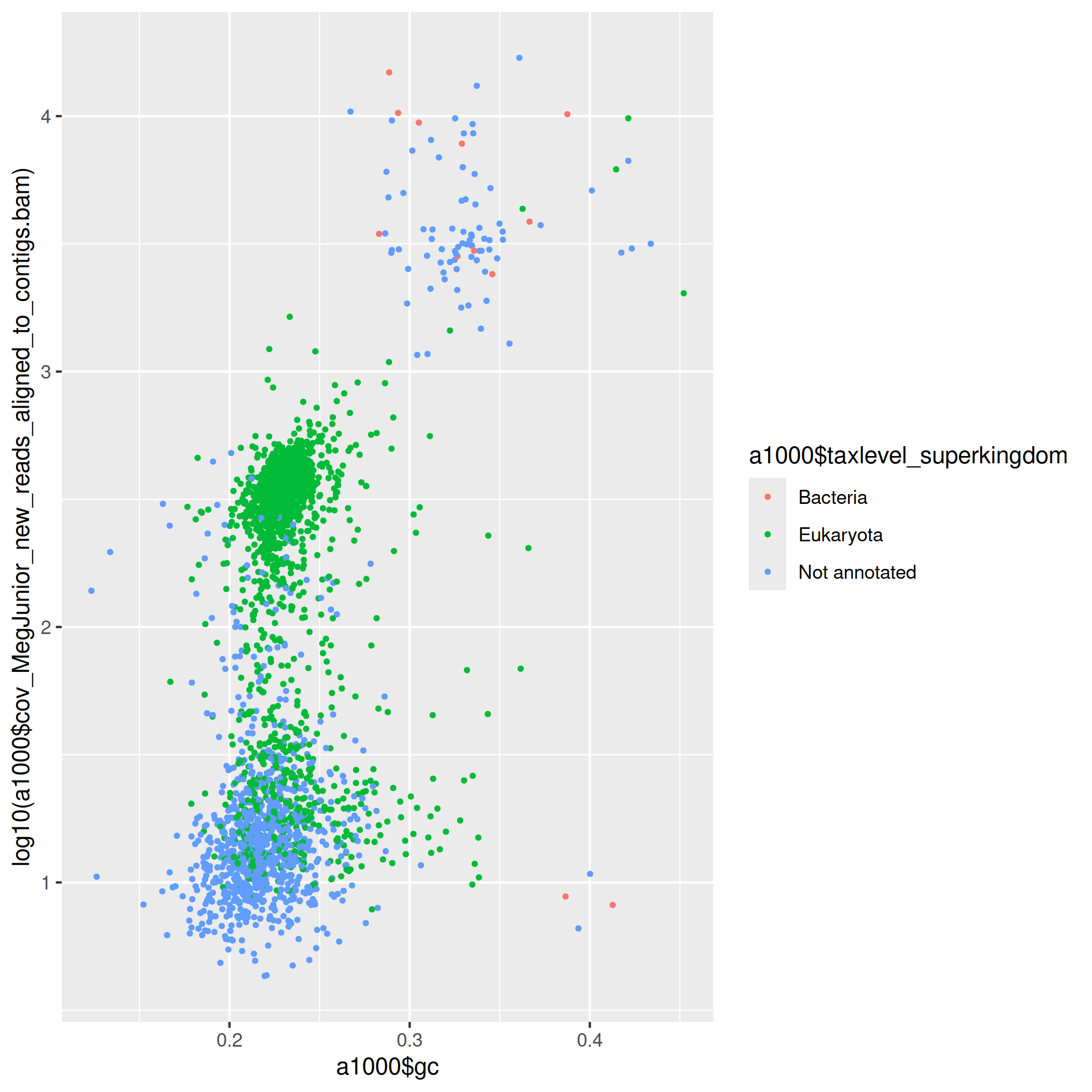

Supplement: Supplementary_material_ycag079 [file supplementary_material_ycag079.zip › Figure S1_blobology_updated.png]
